# Expression of MicroRNAs in the Stem Cell Niche of the Adult Mouse Incisor

**DOI:** 10.1371/journal.pone.0024536

**Published:** 2011-09-08

**Authors:** Andrew H. Jheon, Chun-Ying Li, Timothy Wen, Frederic Michon, Ophir D. Klein

**Affiliations:** 1 Program in Craniofacial and Mesenchymal Biology, Department of Orofacial Sciences, University of California San Francisco, San Francisco, United States of America; 2 Developmental Biology Program, Institute of Biotechnology, University of Helsinki, Helsinki, Finland; 3 Department of Pediatrics and Institute for Human Genetics, University of California San Francisco, San Francisco, United States of America; University of Southern California, United States of America

## Abstract

The mouse incisor is a valuable but under-utilized model organ for studying the behavior of adult stem cells. This remarkable tooth grows continuously throughout the animal's lifetime and houses two distinct epithelial stem cell niches called the labial and lingual cervical loop (laCL and liCL, respectively). These stem cells produce progeny that undergo a series of well-defined differentiation events en route to becoming enamel-producing ameloblasts. During this differentiation process, the progeny move out of the stem cell niche and migrate toward the distal tip of the tooth. Although the molecular pathways involved in tooth development are well documented, little is known about the roles of miRNAs in this process. We used microarray technology to compare the expression of miRNAs in three regions of the adult mouse incisor: the laCL, liCL, and ameloblasts. We identified 26 and 35 differentially expressed miRNAs from laCL/liCL and laCL/ameloblast comparisons, respectively. Out of 10 miRNAs selected for validation by qPCR, all transcripts were confirmed to be differentially expressed. *In situ* hybridization and target prediction analyses further supported the reliability of our microarray results. These studies point to miRNAs that likely play a role in the renewal and differentiation of adult stem cells during stem cell-fueled incisor growth.

## Introduction

Enamel, the outermost layer of teeth and the hardest substance in the mammalian body, is generated by specialized, epithelial-derived cells called ameloblasts. Along with dentin, enamel is one of two mineralized tissues of the tooth crown. Humans possess a limited ability to regenerate enamel due to the loss of ameloblasts upon tooth eruption and the absence of an ameloblast stem cell population. However, some mammals have teeth that grow continuously throughout life. This growth is made possible by the presence of epithelial and mesenchymal stem cells that have the capacity to self-renew and differentiate into ameloblasts and dentin-forming odontoblasts [1]. One such case is the adult mouse incisor, which provides a valuable system for studying the molecular and cellular pathways that govern stem cell self-renewal and differentiation.

**Figure 1:**
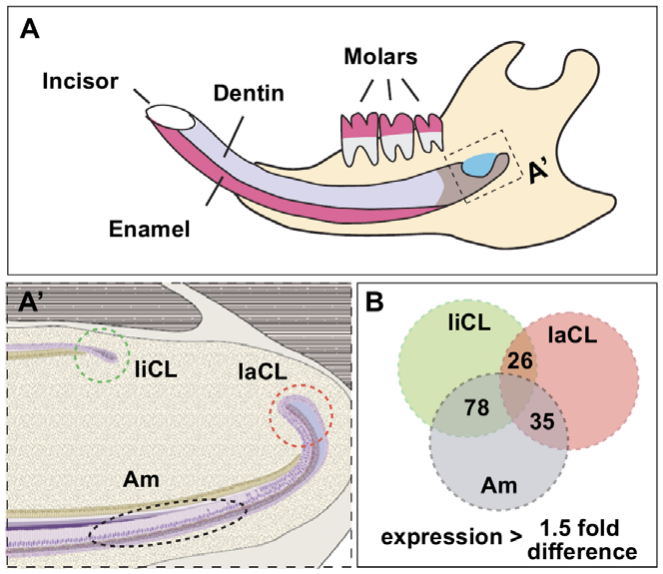
miRNA expression analysis of distinct cell populations in the adult mouse incisor. (A) Cartoon depiction of the adult mouse incisor. (A') Three distinct regions of the adult mouse incisor were isolated for miRNA microarray analysis. liCL, lingual cervical loop; laCL, labial cervical loop; Am, ameloblasts. (B) The number of miRNA transcripts that showed greater than 1.5-fold differential expression (p<0.01) between liCL vs laCL, Am vs laCL, and Am vs liCL are shown. The total number of mouse miRNAs assayed was 458.

Tooth epithelial stem cells reside at the proximal end of the mouse incisor in niches called cervical loops (Fig. 1A) [1]. Previous experiments have shown that epithelial progenitors in the labial cervical loop (laCL) give rise to transit-amplifying (T-A) cells that differentiate into ameloblasts as they migrate distally (Fig. 1A') [1], [2]. The smaller cervical loop on the lingual side (liCL) is also presumed to contain epithelial stem cells, although these cells do not normally give rise to ameloblasts and enamel [3]. Thus, the mouse incisor forms enamel only on the labial surface of the incisor. The mesenchymal compartment between the cervical loops contains the presumptive odontoblast stem cells, which have yet to be characterized (Fig. 1A'). Continuous incisor growth is counterbalanced by abrasion from occlusion of upper and lower incisors and material in the diet [3], [4].

Recent studies indicate that subtle changes in the activity of major signaling pathways, such as those triggered by BMPs, FGFs, and Wnts, can have dramatic effects on incisor growth, thus demonstrating that the precise control of signaling levels is essential for proper generation of enamel [5]. For example, the number of teeth and molar cusp shapes are affected when the BMP, FGF, and Wnt pathways are altered [6]–[11], and changes in levels of the BMP/Activin and FGF signaling pathways affect the size, shape, and mineralization of the incisor [3], [4], [12], [13].

Small RNAs, and miRNAs in particular, have important effects on development and disease through modulation of specific signaling pathways [14]–[16]. miRNAs are endogenously expressed, short (∼21 nucleotides), non-coding RNA molecules that affect protein synthesis by posttranscriptional mechanisms [17], [18]. miRNAs function in the form of ribonucleoproteins called miRISCs (miRNA-inducing silencing complexes), which comprise Argonaute and GW-182 (Glycine-tryptophan (GW) repeat containing protein of 182 kDa) family proteins [19], [20]. miRISCs usually base-pair imperfectly, via component miRNAs, with the 3′UTR of target mRNAs following a set of rules that have been determined using bioinformatics and experimental analyses [21], [22]. This interaction leads to the inhibition of protein synthesis and/or decrease in mRNA stability by disparate mechanisms [19], [20], [23]. The processing of mature miRNAs, of which the most thermodynamically stable are bound to miRISCs, requires several steps. Primary miRNAs (pri-miRNAs) are usually transcribed by RNA polymerase II, folded into single or tandem hairpin structures, and are processed into single hairpins or precursor miRNA (pre-miRNA) by the RNAase III enzyme Drosha in the nucleus [24], [25]. Pre-miRNAs are shuttled into the cytoplasm by Exportin-5 [26], [27] and further processed by a second RNAase III enzyme Dicer [28], [29], which removes the loop part of the duplex, to generate mature miRNAs.

The involvement of miRNAs in various ectodermal tissues has been demonstrated in skin [30], [31], hair [32], and teeth [33]–[36]. During tooth development, the importance of miRNAs was demonstrated by the conditional inactivation of *Dicer* regulated by the expression of Cre recombinase under the *Pitx2* [33] and *K14* [35] promoters. Both *Pitx2* and *K14* are expressed specifically in tooth epithelium. The *Pitx2*-Cre;*Dicer* deleted mice showed a multiplication of the incisors with the absence of enamel, which demonstrated the importance of miRNAs in ameloblast differentiation as well as their role in the regulation of ameloblast stem cells [33]. The *K14*-Cre;*Dicer* deleted mice showed milder changes in tooth shape, epithelial homeostasis, and enamel formation [35].

Here we report the identification and initial characterization of miRNAs that are differentially expressed during stem cell-fueled tooth renewal. By comparing three specific regions in the adult mouse incisor (laCL, which generates ameloblast stem cells; liCL, which contains stem cells that do not normally give rise to ameloblasts; and ameloblasts), we have identified miRNAs that may play a role in the self-renewal and differentiation of stem cells.

## Results

### Identification of differentially expressed miRNAs

The adult mouse hemi-mandible contains a continuously growing incisor and three molar teeth (Fig. 1A). Although specific molecular pathways involved in tooth development have been well documented, little is known regarding the role of miRNAs in this process. Therefore, we set out to identify miRNAs that could be involved in the renewal and differentiation of ameloblast stem cells. We isolated three regions from the incisor (Fig. 1A'): the laCL, which comprises ameloblast stem cells; the liCL, which houses stem cells that do not normally give rise to ameloblasts and enamel; and pre- and secretory ameloblasts, which produce enamel.

**Figure 2:**
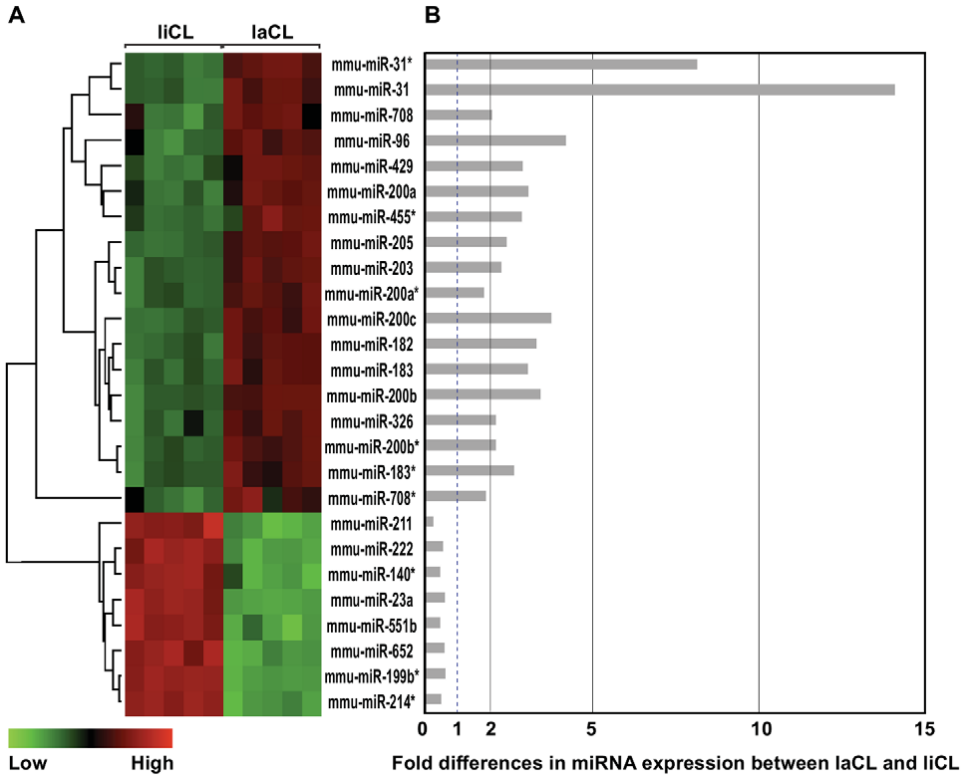
Differentially expressed miRNAs between laCL and liCL. (A) Heat map of miRNAs that are differentially expressed 1.5-fold (p<0.01) between laCL and liCL. (B) Bar graph showing fold changes.

**Figure 3:**
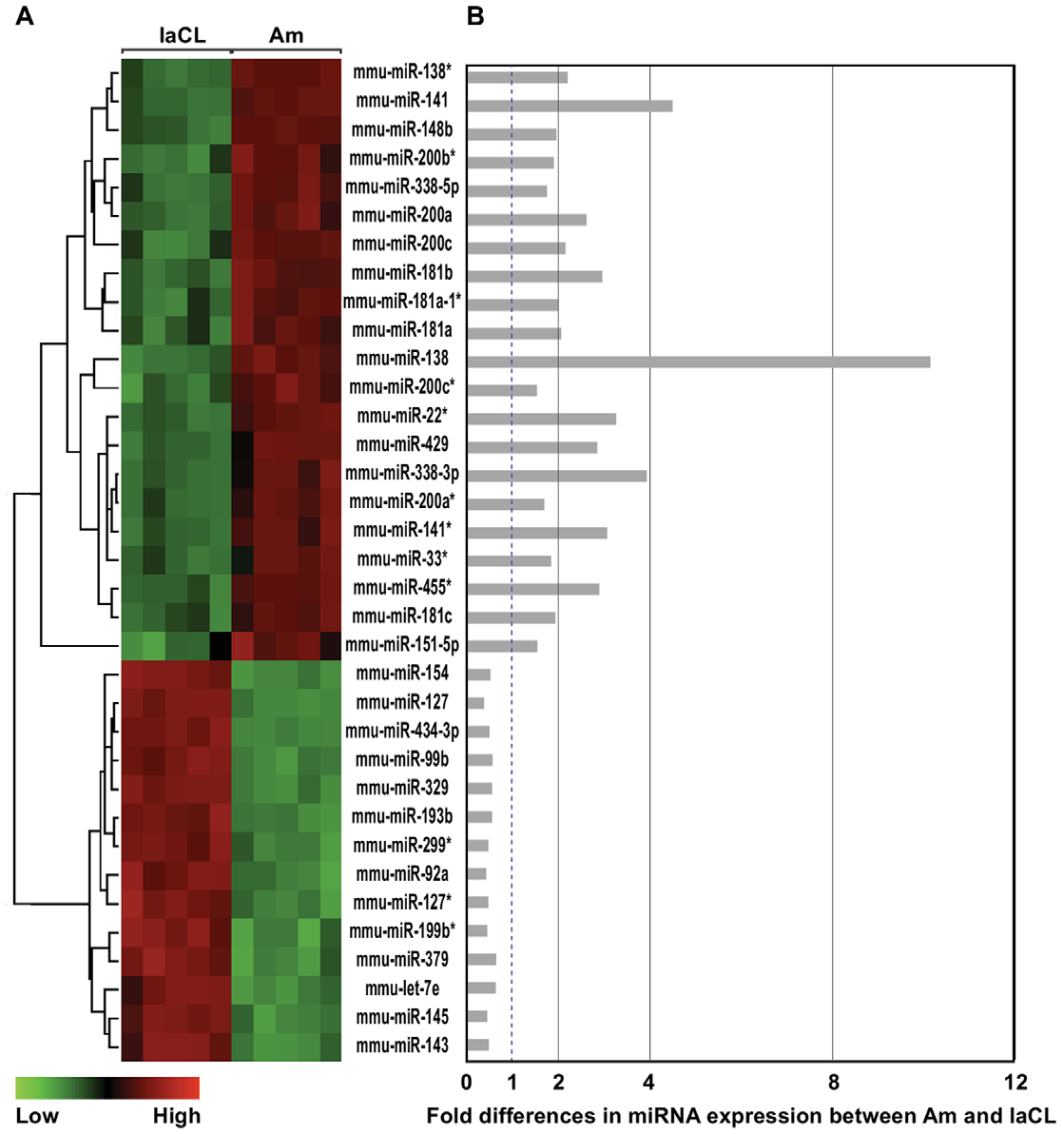
Differentially expressed miRNAs between Am and laCL. (A) Heat map of miRNAs that are differentially expressed 1.5-fold (p<0.01) between Am and laCL. (B) Bar graph showing fold changes.

Regions containing these cells, which are readily visible in *K14*-eGFP mice, were isolated by microdissection and then miRNA microarray analysis was performed. From a total of 458 mouse miRNAs assayed, we identified transcripts that were differentially expressed by more than 1.5-fold with adjusted p-values of less than 0.01 in the following pairwise comparisons: laCL/liCL, ameloblasts/laCL, and ameloblasts/liCL (Fig. 1B). Heat maps and fold-differences of the differentially expressed miRNAs in laCL/liCL (Fig. 2A,B) and ameloblasts/laCL (Fig. 3A,B) comparisons were produced. Many differentially expressed miRNAs were miRNA* (i.e. star strand) species, which are not well characterized.

We reasoned that the laCL/liCL comparison would highlight the miRNAs involved in the renewal of ameloblast stem cells, whereas the ameloblast/laCL comparison would identify miRNAs involved in the progression of stem cells and their progeny towards terminal differentiation. miRNAs from the ameloblast/liCL comparison were not pursued for further study. Information about the differentially expressed miRNAs identified from laCL/liCL and ameloblasts/laCL comparisons is summarized in Tables S1 and S2 along with the relevant references in Supplemental References S1. With the exception of the miR-200 family (i.e. miR-141, -200a, -200b, -200c, -429) and miR-199b*, there was very little overlap in the differentially expressed miRNAs identified from laCL/liCL and ameloblasts/laCL comparisons.

### Confirmation of differentially expressed miRNAs by real-time qPCR

**Figure 4:**
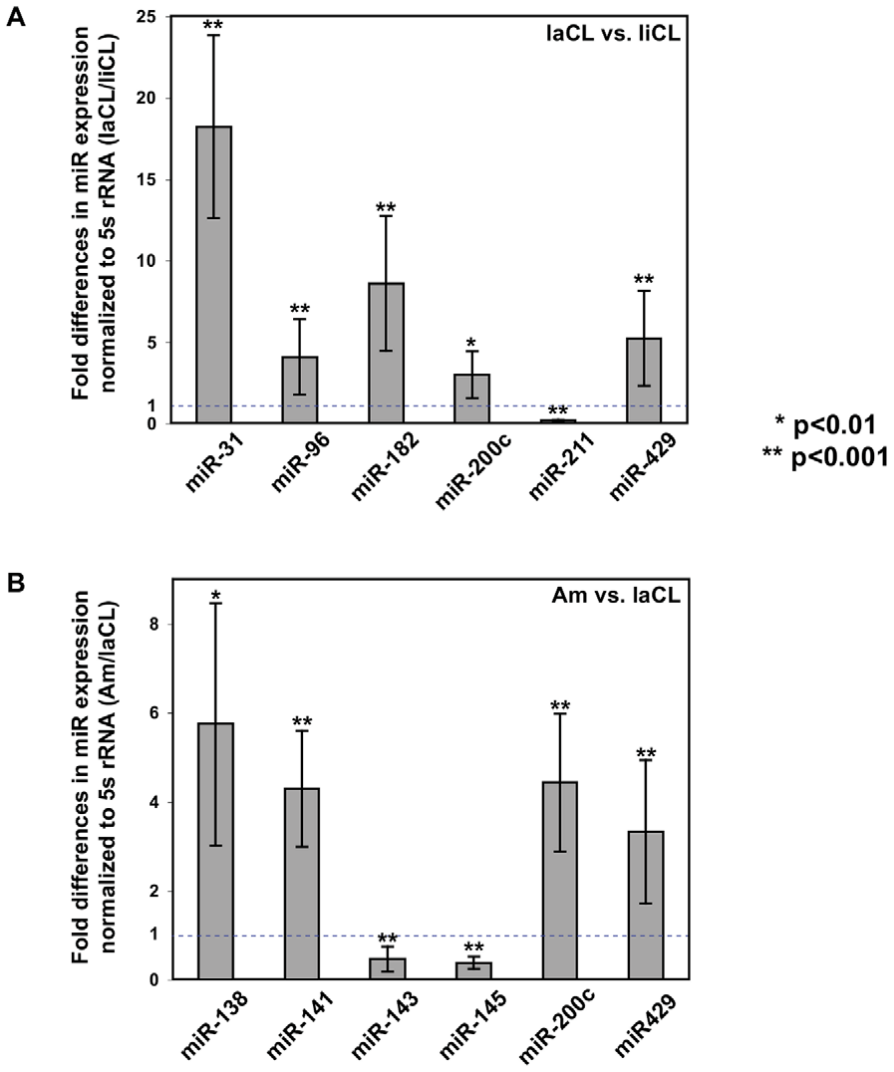
qPCR confirmation of differentially expressed miRNAs. (A,B) Fold expression differences of miRNAs identified from the laCL/liCL (A) and ameloblast/laCL (B) comparisons measured by qPCR. Expression levels were normalized to 5s rRNA.

From the differentially expressed miRNAs identified from laCL/liCL (Fig. 2) and ameloblasts/laCL (Fig. 3) comparisons, we selected 6 miRNAs from each comparison for confirmation by qPCR. The miRNAs were chosen based on high differential expression and included both those with increases and decreases in expression fold-differences. All the miRNAs assayed by qPCR were confirmed to be differentially expressed (Fig. 4), which validated the microarray approach. Thus, the array results for all differentially expressed miRNAs are expected to be accurate. From the laCL/liCL comparison, miR-31, -96, -182, -200c, -429 levels were increased, and miR-211 levels were decreased, in the laCL region compared to the liCL region (Fig. 4A). miR-138, -141, -200c, -429 were confirmed to be expressed highly, whereas miR-143, -145 levels were less abundant, in ameloblasts compared to the laCL region (Fig. 4B).

### Locailization of miR-31 and miR-138 expression

**Figure 5:**
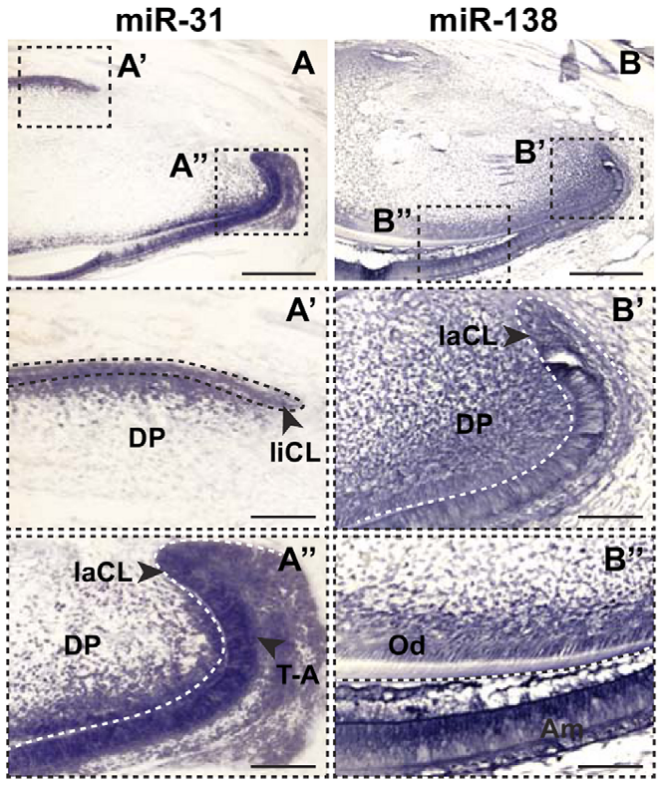
*In situ* hybridization analysis of miR-31 and miR-138. (A,B) Localization of miR-31 (A) and miR-138 (B) expression in the mouse adult incisor. Scale bars, 200 µm. (A',A″) Magnified views of miR-31 expression in the liCL (A') and laCL (A″) regions. (B,B″) Magnified views of miR-138 expression in the laCL region (B') and in the ameloblasts (Am, B″). Scale bars, 50 µm. Am, ameloblasts; DP, dental papilla; Od, odontoblasts; T-A, transit-amplifying cells.

miR-31 showed 14 to 18-fold higher expression in the laCL region compared to the liCL (Figs. 2, 4) and miR-138 showed 6 to 10-fold higher expression in ameloblasts compared to the laCL (Fig. 3, 4). *In situ* hybridization was performed on these two most differentially expressed miRNAs to determine their localization in the adult mouse incisor. miR-31 localized largely to the laCL, specifically in the region of the T-A cells, although there was also expression in the mesenchyme-derived dental papilla adjacent to the laCL and liCL (Fig. 5A). miR-138 was expressed in the laCL and ameloblasts, as well as in the dental papilla and odontoblasts, and miR-138 expression appeared to be higher in ameloblasts than in the laCL (Fig. 5B).

### Predicting gene targets

For each differentially expressed, array-identified miRNA, the predicted targets were retrieved from miRTooth1.0 database (Tables S3, S4) (http://bite-it.helsinki.fi/miRNA.htm). Only the predicted targets found in at least 2 out of 3 prediction databases were retained, similar to the strategy we have previously used [35].

Bioinformatic target analysis demonstrated the validity of the microarray experiments on a larger scale than the focused qPCR confirmations and *in situ* hybridization analyses. First, the miRNAs enriched in each region (laCL, liCL or ameloblasts) were predicted to target distinct sets of genes (Tables S3, S4). For example, 67% of the miRNAs in ameloblasts, compared to 9% of miRNAs in the laCL, target components of the FGF pathway, an important pathway in both tooth development and in the homeostasis of the stem cell niche [37], [38]. In addition, Sprouty2 (*Spry2*), an antagonist of FGF signaling, is a potential target of miRNAs enriched in the laCL from all 3 databases. Second, laCL and liCL populations, but not ameloblasts, are enriched for miRNAs that target amelogenin and ameloblastin, two essential matrix proteins in enamel development in mice and humans [39], [40]. Third, the regulation of extracellular matrix components appears to be more extensive in ameloblasts, which secrete enamel matrix, compared to the laCL (58% vs. 18%, respectively).

## Discussion

The continuously growing mouse incisor provides a valuable model system to study adult stem cells. A single incisor contains two different epithelial stem cell niches, called the laCL and liCL, which are both derived from oral epithelium and surrounded by dental mesenchyme. Of these two niches, only the laCL generates enamel-forming ameloblasts. Therefore, we hypothesized that molecular profiling of the laCL and liCL, as well as the ameloblast region, would shed light on the renewal and differentiation of ameloblast stem cells.

miRNAs are important regulators of signaling pathways during morphogenesis and organogenesis, as well as in the control of embryonic and adult stem cells [41]. The importance of *Dicer* and miRNAs during tooth development has been demonstrated [16], [33], [35], but there has been relatively little progress in the identification and characterization of the roles of specific miRNAs. Such molecular characterization of dental stem cells may one day help us to repair and regenerate human teeth.

The miRNA microarray analysis from laCL, liCL, and ameloblasts yielded numerous differentially expressed miRNA transcripts (Figs. 2, 3). All 10 miRNAs that we chose for validation by qPCR were indeed differentially expressed in the laCL/liCL and ameloblast/laCL comparisons at levels consistent with the microarray data (Fig. 4). Because the laCL, liCL, and ameloblast regions isolated from the incisor included mesenchyme-derived tissues, we selected two of the highest differentially expressed genes (i.e. miR-31, -138) for *in situ* hybridization analysis. Our results showed high expression of miR-31 in the laCL and miR-138 in ameloblasts, consistent with the microarray and qPCR analyses.

miR-31 was previously reported to be involved in the cycling of ectoderm-derived hair cells [42]. Because the hair stem cell niche shares many characteristics with the laCL [4], [43], it is likely that miR-31 will also play a role in the renewal of stem cells during continuous incisor growth. Interestingly, *Fgf10* was previously identified as a direct target of antagonism by miR-31 [42]. *Fgf10* is expressed largely in the dental papilla adjacent to the laCL [37], [38], [44]. *Fgf10* is required for tooth stem cell survival [38], and its inactivation leads to the formation of smaller teeth [4], [38], whereas upregulation of *Fgf10* is associated with the generation of supernumerary teeth and ectopic enamel [3], [6]. Taking into account the high expression of miR-31 in the ectoderm-derived laCL, it is possible that miR-31 may play a role in fine-tuning *Fgf10* levels in the dental papilla. Further experiments will be required to study the interaction between miR-31 and *Fgf10* during continuous tooth growth.

miR-138, whose expression is correlated with many different types of cancers and diseases, is also involved in cardiac patterning, specifically through the regulation of expression of genes such as aldehyde dehydrogenase (*Aldh*) -1a2 and versican [45]. Because *Aldh1* is a marker of stem cells in certain contexts [46] and versican is a secreted extracellular matrix protein that is present in many mineralized tissues including the dental epithelium of developing tooth germs [47], miR-138 may be involved in the differentiation of stem cells towards enamel matrix-secreting ameloblasts.

Members of the miR-200 family (i.e. miR-200a, -200b, -200c, -141, -429) were differentially expressed in both laCL/liCL and laCL/ameloblast comparisons. These transcripts along with miR-199b* were the only miRNAs that were differentially expressed in both comparisons, demonstrating the potential importance of specific subsets of miRNAs in the renewal vs. differentiation of tooth stem cells.

Interestingly, many of the microarray-identified, differentially expressed miRNAs were miRNA* (i.e. star strand) species. The ∼21-nucleotide, obligate intermediate miRNA:miRNA* duplex associates with the Argonaute protein in miRISCs, such that the miRNA strand is usually the one that becomes stably incorporated, whereas the miRNA* strand dissociates and is thought to be degraded [48]. Thus, by convention, the mature miRNA is defined as the duplex strand that is present at the higher steady-state level than its miRNA* partner strand. However, Yang et al. [48] recently demonstrated that miRNA* species, rather than simply being the by-product of non-incorporation into miRISCs, also could directly repress translation and/or mRNA stability by specific binding to the 3′-UTR of genes. Further, the target seed sequence, which is a conserved heptametrical sequence that is essential for the binding of the miRNA to the mRNA, was evolutionarily conserved in miRNA* species [48]. Thus, it will be of interest to determine the function of the identified miRNA* species during stem cell renewal and differentiation.

Together, our analyses utilizing microarray technology, qPCR, *in situ* hybridization, and target prediction tools have uncovered miRNAs that may play important roles in stem cell-based renewal of teeth.

## Materials and Methods

### Animals and isolation of tissues

*K14*-eGFP transgenic mice [49] were maintained and genotyped as previously reported. All experiments were approved by the Institutional Animal Care and Use Committee (IACUC) at the University of California San Francisco (Protocol # AN078624). Six-week old male mice were sacrificed, and the laCL, liCL, and ameloblast regions were microdissected under a LeicaMZ16F Fluorescence Stereomicroscope.

### RNA extraction

miRNAs were extracted using the miRNeasy kit (Qiagen) following manufacturer's protocol.

### Histology

Heads from 6-week old mice were fixed for 48 h in 4% paraformaldehyde at 4°C, cut in half along the midline, demineralized in 0.5M EDTA for 2 weeks, dehydrated, embedded in paraffin wax, and serially sectioned at 7 µm.

### Microarray and differential expression analysis

Probe labeling and array hybridizations were performed according to standard protocols from the UCSF Shared Microarray Core Facilities and Agilent Technologies (http://www.arrays.ucsf.edu and http://www.agilent.com). miRNA quality was assessed using a Pico Chip on an Agilent 2100 Bioanalyzer (Agilent Technologies, Palo Alto, CA). RNA was amplified and labeled with Cy3-CTP using the Agilent low RNA input fluorescent linear amplification kits following the manufacturer's protocol (Agilent). Labeled cRNA was assessed using the Nandrop ND-100 (Nanodrop Technologies, Inc., Wilmington DE), and equal amounts of Cy3 labeled target were hybridized to custom v3.1 multi-species 8×15k miRNA arrays (Agilent). Hybridizations were performed for 14 hours, according to the manufacturer's protocol. Arrays were scanned using a microarray scanner and raw signal intensities were extracted with Feature Extraction v10.3 software (Agilent).

Raw log-intensities were normalized using the *quantile* normalization method proposed by Bolstad *et al*. [50]. No background subtraction was performed, and the median feature pixel intensity was used as the raw signal before normalization. A two-way ANOVA model and specific contrasts were formulated to examine comparisons between treatments (wild-type versus *Perp*-null). Moderated t-statistic, B statistic, false discovery rate, and p-value for each gene were obtained. Adjusted p-values were produced by the method proposed by Holm [51]. All procedures were carried out using functions in the “R” package *limma in Bioconductor* [52], [53]. Heat maps were generated using Cluster 3.0 and MapleTree 12.0.0.

All data is MIAME compliant and raw data has been deposited in the Gene Expression Omnibus (GEO) database with accession number GSE30598.

### Real-time qPCR

cDNA was generated from miRNA using the miRCURY Universal cDNA synthesis kit (Exiqon). qPCR analysis was performed using Locked Nucleic Acid (LNA) primer sets (Exiqon) specific for miR-31, -96, -138, -141, -143, -145, -182, 200c, -211, -429, 5s rRNA and the SBYR Green Master Mix (Exiqon). qPCR reaction conditions were as follows: 95°C, 10 min; 40 cycles of 95°C, 10 s and 60°C, 1 min. Levels of miRNA were normalized to 5s rRNA (Exiqon).

### *In situ* hybridization

*In situ* hybridization was performed on paraffin sections essentially as described [6] except that DIG-labeled LNA probes specific for miR-31 and miR-138 (Exiqon) were hybridized using microRNA ISH buffer (Exiqon).

### miRNA target genes prediction

Putative target genes for selected miRNAs were predicted using the miRTooth1.0 database (http://bite-it.helsinki.fi/miRNA.htm) as described previously [35].

### Statistical analysis

All experiments were performed independently at least three times in triplicate, and when applicable, presented as an average ± standard deviation. Except for the microarray analysis, the Student t-test was used to determine p-values and p<0.01 was deemed to be significant.

## Supporting Information

Table S1 **Summary of differentially expressed miRNAs in laCL and liCL.**
(PDF)

Table S2 **Summary of differentially expressed miRNAs in Am vs laCL.**
(PDF)

Table S3 **Predicted targets of differentially expressed miRNAs identified from the laCL/liCL comparison.**
(PDF)

Table S4 **Predicted targets of differentially expressed miRNAs identified from the ameloblast/laCL comparison.**
(PDF)

Supplemental References S1 (PDF)
