# Supplementary material for: Expression of MicroRNAs in the Stem Cell Niche of the Adult Mouse Incisor
Source: PLoS One. 2011 Sep 8;6(9):e24536. doi: 10.1371/journal.pone.0024536 (PMC3169592; doi:10.1371/journal.pone.0024536)
Supplement: Table S1 — Summary of differentially expressed miRNAs in laCL and liCL. (PDF) [file pone.0024536.s001.pdf]

Table S1. Summary of differentially expressed miRNAs in laCL and liCL.

Symbol	Function	References
miR-31*	Unknown function.	
miR-31	miR-31 controls hair cycle-related changes and negatively regulates expression of Fgf10. miR-31 also acts as an oncogenic miRNA (oncomir) in lung cancer by targeting specific tumor suppressors for repression.	[1,2]
miR-708*	Unknown function.	
miR-96	Member of the miR-183~182 cluster. Cluster comprises miR-96, -182, and -183. miR-96 suppresses KRAS and functions as a tumor suppressor in pancreatic cancer.	[3]
miR-429	Member of the miR-200 family (i.e. miR-200a, 200b, 200c, 141, and 429). miR-200 family members regulate epithelial-mesenchymal transition (EMT) by downregulating ZEB1 and ZEB2, which are repressors of E-cadherin. Overexpression of miR-429 leads to mesenchymal to epithelial transition in metastatic ovarian cancer cells.	[4-7]
miR-200a	Member of the miR-200 family. See miR-429. Unknown function.	
miR-455*	Unknown function.	
miR-205	Over-expression of miR-205 leads to decreased cell size and increased proliferation in mammary epithelial cell progenitors. miR-205 targets include PTEN, ZEB1, and ZEB2.	[5,8]
miR-203	miR203 regulates p63, a member of the p53 family that regulates morphogenesis of ectodermal derivatives such as skin, hair, and teeth. Overexpression of miR-203 leads to decreased clonogenic capacity of skin stem cells in culture, restricts proliferation, and induces cell cycle exit.	[9,10]
miR-200a*	Member of the mIR-200 family. Unknown function.	
miR-200c	Member of the miR-200 family. See miR-429. miR-200c regulates the induction of apoptosis by targeting FAP. Along with miR141, inhibits JAG1.	[7,11]
miR-182	Member of the miR-183~182 cluster. Cluster comprises miR-96, -182, and -183. mIR-182 mediates the downregulation of BRCA1 and impairs DNA repair. Also, the specific inhibition of miR-182 in helper T lymphocytes limits their population expansion *in vivo* and *in vitro*.	[12,13]
miR-183	Member of the miR-183~182 cluster. Cluster comprises miR-96, -182, and -183. miR-183 targets integrin β1 (*ITGB1*) and kinesin 2α (*KIF2A*), and may be involved in the development and function in neurosensory organs.	[14]
miR-200b	Member of the miR-200 family. See miR-429. The miR-200b-Suz12-E-cadherin pathway is important for cancer stem cell growth and its transcriptional signature is observed in metastatic breast tumors.	[15]
miR-326	miR-326 regulates interleukin-17 producing T-helper cells and is involved in the pathogenesis of multiple sclerosis.	[16]
miR-200b*	Member of the mIR-200 family. Unknown function.	
miR-183*	Unknown function.	
miR-708*	Unknown function.	
miR-211	miR-211 is a suppressor of melanoma invasion. Also, the over-expression of miR-211 increased proliferation, migration, and anchorage-independent colony formation of oral carcinoma cells.	[17,18]
miR-222	miR-221/222 targets the tumor suppressor, p27/Kip1, and affects proliferative potential of various cells.	[19,20]
miR-140*	Unknown function.	
miR-23a	The miR-23a~27a~24-2 cluster is downregulated by Runx2 and the cluster represses translation of Runx2 and Satb2 during osteoblast differentiation.	[21]
miR-551b	Unknown function.	
miR-652	Unknown function.	
miR-199b*	Unknown function.	
miR-214*	Unknown function.	
