# Supplementary material for: Expression of MicroRNAs in the Stem Cell Niche of the Adult Mouse Incisor
Source: PLoS One. 2011 Sep 8;6(9):e24536. doi: 10.1371/journal.pone.0024536 (PMC3169592; doi:10.1371/journal.pone.0024536)
Supplement: Table S2 — Summary of differentially expressed miRNAs in Am vs laCL. (PDF) [file pone.0024536.s002.pdf]

Table S2. Summary of differentially expressed miRNAs in Am vs laCL.

Symbol	Function	References
miR-138*	Unknown function.	
miR-141	Member of the miR-200 family (i.e. miR-200a, 200b, 200c, 141, and 429). Along with miR-200c, inhibits JAG1.	[7]
miR-148b	Unknown function.	
miR-200b*	Member of the mIR-200 family. Unknown function.	
miR-338-3p	miR-338-3p and miR-451 are necessary for the development of epithelial cell polarity.	[22]
miR-200a	Member of the mIR-200 family. See Table 1.	
miR-200c	Member of the mIR-200 family. See Table 1.	
miR-181b	Over-expression of miR-21, miR-181b and miR-345 is associated with the progression of leukoplakia to oral carcinoma.	[23]
miR-181a-1*	Unknown function.	
miR-181a	miR-181a is downregulated by phospho (p)-ΔNp63α; downregulates K-ras and shows tumor suppressive effects against oral squamous cell carcinoma cells; and is an intrinsic modulator or T cell sensitivity and selection.	[24-26]
miR-138	miR-138 is required for the proper expression patterns of genes such as aldehyde dehydrogenase-1a2 and versican in zebrafish heart.	[27]
miR-200c*	Member of the mIR-200 family. Unknown function.	
miR-22*	Unknown function.	
miR-429	Member of the mIR-200 family. See Table 1.	
miR-338-5p	Unknown function.	
miR-200a*	Member of the mIR-200 family. Unknown function.	
miR-141*	Unknown function.	
miR-33*	Unknown function.	
miR-455*	Unknown function.	
miR-181c	miR-181c targets interleukin-2 in activated CD4(+) T lymphocytes.	[28]
miR-151-5p	miR-151-5p increases hepatocellular carcinoma (HCC) cell migration and invasion in vivo and in vitro. miR-151-5p directly targets RhoGDIA, a putative metastasis suppressor in HCC, leading to the activation of Rac1, Cdc42 and Rho GTPases.	[29]
miR-154	Unknown function.	
miR-127	Member of the miR-127~433 cluster. Unknown function.	
miR-434-3p	Unknown function.	
miR-99b	Unknown function.	
miR-329	Member of the miR379-410 cluster. Cluster comprises 50 miRNAs. Unknown function.	
miR-193b	miR-193b represses cell proliferation during melanoma development.	[30]
miR-299*	Unknown function.	
miR-92a	Member of the miR-17-92 cluster. Cluster comprises miR-17-5p and -3p, miR-18a, miR-19a and b, miR-20a and miR-92a. Unknown function.	
miR-127*	Unknown function.	
miR-199b*	Unknown function.	
miR-379	Unknown function.	
let-7e	Although let-7e has not yet been studied, let-7 family members target many genes important for stem cells, including *Hmga2*.	[31,32]
miR-145	Member of the miR-143-145 cluster. miR-145 regulates OCT4, SOX2, and KLF4 and represses pluripotency in human embryonic stem cells; inhibits cell proliferation of human lung adenocarcinoma by targeting EGFR and NUDT1. miR-143-145 cluster plays a critical role during smooth muscle cell differentiation, and is repressed by KRAS to initiate a tumor-promoting pathway.	[33-36]
miR-143	Member of the miR-143-145 cluster. miR-143-145 cluster plays a critical role during smooth muscle cell differentiation, and is repressed by KRAS to initiate a tumor-promoting pathway.	[35,36]
