# Supplementary material for: Expression of MicroRNAs in the Stem Cell Niche of the Adult Mouse Incisor
Source: PLoS One. 2011 Sep 8;6(9):e24536. doi: 10.1371/journal.pone.0024536 (PMC3169592; doi:10.1371/journal.pone.0024536)
Supplement: Table S3 — Predicted targets of differentially expressed miRNAs identified from the laCL/liCL comparison. (PDF) [file pone.0024536.s003.pdf]

Table S3. Predicted targets of differentially expressed miRNAs identified from the laCL/liCL comparison.

**Symbol**	**Predicted target genes** (2 databases, 3 databases)
miR-31*	
miR-31	aquaporin4 *Dlx4* *Tfap2a* *Axin1* *Odc*
miR-708*	
miR-96	*Dlx2* integrin b1 *Msx2* *Runx1* *Tgfb2* *Tip2* *Mmp2* *Tbx1*
miR-429	fibronectin kallikrein4 *Pitx2* reelin *Ror2* *Sema3f* *Snai1*
miR-200a	*Fgf7* *Mme* *Mmp13* notch1 *Runx1* *Dlx5* *Pigpen* *Pitx2*
miR-455*	
miR-205	*Arnt* *Axin2* *Dlx6* *Mmp2* *Pax9* *Tgfb3* *Traf3* activin bA
miR-203	*Barx1* *Col4a1* *Dlx5* *Fgf7* *Gli3* *Hand2* integrin av tenascin C versican ectodin
miR-200a*	
miR-200c	fibronectin kallikrein4 occludin phosphacan *Snai1* reelin *Ror2*
miR-182	*Epha7* *Hand1* laminin a1 *Oasis* *Tbx1* *Rarg*
miR-183	integrin a6 jagged1 *Msx1* notch1 *Plu1* *Slitrk6* integrin b1 *Spry2*
miR-200b	fibronectin kallikrein4 phosphacan reelin *Ror2* *Slit2* *Snai1*
miR-326	*Axin2* *Bmp4* clusterin ectodin *Gli2* *Hspg2* *Irx2* midkine notch3 plakoglobin *Rarg* *Ror2* *Runx1* *Tfap2c* *Tlx1* activin bA neuropilin1 tenascin C
miR-200b*	
miR-183*	
miR-708*	
miR-211	*Bmp7* cerebroglycan *Dlx1* *Dlx4* *Egr1* *Fgf8* *Hspg2* *Mmp13* plakoglobin *Pvrl1* *Tfap2c*
miR-222	ameloblastin *Dlx1* *Gfra1* *Irx5* jagged1 *Lhx8* reelin *Sema3b*
miR-140*	
miR-23a	ameloblastin *Bmp3* *Ikka* *Pax9* *Runx1* *Rxrg* *Tip2*
miR-551b	*Bmp5* integrin b4 *Sp6* *Spry1* *Tgfb3*
miR-652	*Cspg4* integrin av islet1 *Runx1* *Tgfb3*
miR-199b*	
miR-214*	
