# Supplementary material for: Expression of MicroRNAs in the Stem Cell Niche of the Adult Mouse Incisor
Source: PLoS One. 2011 Sep 8;6(9):e24536. doi: 10.1371/journal.pone.0024536 (PMC3169592; doi:10.1371/journal.pone.0024536)
Supplement: Table S4 — Predicted targets of differentially expressed miRNAs identified from the ameloblast/laCL comparison. (PDF) [file pone.0024536.s004.pdf]

Table S4. Predicted targets of differentially expressed miRNAs identified from the ameloblast/laCL comparison.

**Symbol**	**Predicted target genes** (2 databases, 3 databases)
miR-138*	
miR-141	*Dlx5* fibronectin *Mme* notch1 pigpen *Pitx2* *Rarb* *Runx1* *Slitrk6*
miR-148b	*Axin1* *Col4a1* integrin a6 k-glypican notch3 tenascin *Tgfb3* tuftelin *Wnt10a* *Hes1* *Slit1*
miR-200b*	
miR-338-3p	*Fgf7* *Fgf8* *Gli2* integrin a6 *Oasis* *Osr2* *Plu1* *Spry2* *Tac1* tenascin C
miR-200a	*Fgf7* *Mme* *Mmp13* *notch1* *Runx1* *Dlx5* pigpen *Pitx2*
miR-200c	fibronectin kallikrein4 occludin phosphacan *Snai1* reelin *Ror2*
miR-181b	*Col4a1* *Fgfr2* *Hand2* *Igf1* *Mmp14* *Pitx2* *Traf1* *Fgf7* *Hgf*
miR-181a-1*	
miR-181a	*Fgf7* *Fgfr2* *Hand2* jagged1 *Traf1* *Hgf* *Igf1* *integrin a6* *Mmp14*
miR-138	*Col4a1* *Fgfr2* *Hand2* *Oasis* *Osr2* *Rara* *Wnt4* *Wnt6* *Axin1*
miR-200c*	
miR-22*	
miR-429	fibronectin kallikrein4 *Pitx2* reelin *Ror2* *Sema3f* *Snai1*
miR-338-5p	*Fgfr3* neuropilin1 *Tgfb3* *Traf1* *Wnt4*
miR-200a*	
miR-141*	
miR-33*	
miR-455*	
miR-181c	*Bmp3* *c-MyB* *Col4a1* *Hand2* *Hgf* integrin a6 *Traf1* *Fgf7* *Fgfr2* *Mmp14*
miR-151-5p	*Fgfr4* *Irx4*
miR-154	*Eda* integrin a6
miR-127	integrin a6 integrin b5 *Osr2* *Rxrb* *Slit1* *Pitx2*
miR-434-3p	*Dlx5* integrin av *Ror2* *Tip1*
miR-99b	*Hgf* *Tfap2a*
miR-329	*Arnt* integrin b5 *Met* *Pax9* pigpen plakoglobin *Rxrg* *Traf3* *Axin1* *Mmp14*
miR-193b	*Irx2* *Mmp14* plakoglobin
miR-299*	
miR-92a	*Hand1* *Hand2* integrin a6
miR-127*	
miR-199b*	
miR-379	fibronectin *Mmp14* *Rarb* tuftelin *Rxrb* *Sema3a*
let-7e	amelogenin aquaporin4 *Hand1* *Tfap2a*
miR-145	aquaporin4 *Dermo1* *Epha7* *Gli3* *Rxra* *Sema3a* *Slitrk6* *Traf1* *Traf3*
miR-143	*Arnt* *Axin1* *Bmp5* *Ctgf* *Dermo1* *Gli3* *Tgfb2* *Wnt10a* *Fgf7*
