# Supplementary material for: Expression of MicroRNAs in the Stem Cell Niche of the Adult Mouse Incisor
Source: PLoS One. 2011 Sep 8;6(9):e24536. doi: 10.1371/journal.pone.0024536 (PMC3169592; doi:10.1371/journal.pone.0024536)
Supplement: Supplemental References S1 — (PDF) [file pone.0024536.s005.pdf]

Supplemental References

1. Mardaryev AN, Ahmed MI, Vlahov NV, Fessing MY, Gill JH, et al. (2010) Micro-RNA-31 controls hair cycle-associated changes in gene expression programs of the skin and hair follicle. FASEB J 24: 3869-3881.
2. Liu X, Sempere LF, Ouyang H, Memoli VA, Andrew AS, et al. (2010) MicroRNA-31 functions as an oncogenic microRNA in mouse and human lung cancer cells by repressing specific tumor suppressors. J Clin Invest 120: 1298-1309.
3. Yu S, Lu Z, Liu C, Meng Y, Ma Y, et al. (2010) miRNA-96 suppresses KRAS and functions as a tumor suppressor gene in pancreatic cancer. Cancer Res 70: 6015-6025.
4. Chen J, Wang L, Matyunina LV, Hill CG, McDonald JF (2011) Overexpression of miR-429 induces mesenchymal-to-epithelial transition (MET) in metastatic ovarian cancer cells. Gynecol Oncol.
5. Gregory PA, Bert AG, Paterson EL, Barry SC, Tsykin A, et al. (2008) The miR-200 family and miR-205 regulate epithelial to mesenchymal transition by targeting ZEB1 and SIP1. Nat Cell Biol 10: 593-601.
6. Park SM, Gaur AB, Lengyel E, Peter ME (2008) The miR-200 family determines the epithelial phenotype of cancer cells by targeting the E-cadherin repressors ZEB1 and ZEB2. Genes Dev 22: 894-907.
7. Vallejo DM, Caparros E, Dominguez M (2011) Targeting Notch signalling by the conserved miR-8/200 microRNA family in development and cancer cells. EMBO J.
8. Greene SB, Gunaratne PH, Hammond SM, Rosen JM (2010) A putative role for microRNA-205 in mammary epithelial cell progenitors. J Cell Sci 123: 606-618.
9. Lena AM, Shalom-Feuerstein R, Rivetti di Val Cervo P, Aberdam D, Knight RA, et al. (2008) miR-203 represses 'stemness' by repressing DeltaNp63. Cell Death Differ 15: 1187-1195.
10. Yi R, Poy MN, Stoffel M, Fuchs E (2008) A skin microRNA promotes differentiation by repressing 'stemness'. Nature 452: 225-229.
11. Schickel R, Park SM, Murmann AE, Peter ME (2010) miR-200c regulates induction of apoptosis through CD95 by targeting FAP-1. Mol Cell 38: 908-915.
12. Moskwa P, Buffa FM, Pan Y, Panchakshari R, Gottipati P, et al. (2011) miR-182-Mediated Downregulation of BRCA1 Impacts DNA Repair and Sensitivity to PARP Inhibitors. Mol Cell 41: 210-220.
13. Stittrich AB, Haftmann C, Sgouroudis E, Kuhl AA, Hegazy AN, et al. (2010) The microRNA miR-182 is induced by IL-2 and promotes clonal expansion of activated helper T lymphocytes. Nat Immunol 11: 1057-1062.
14. Li G, Luna C, Qiu J, Epstein DL, Gonzalez P (2010) Targeting of integrin beta1 and kinesin 2alpha by microRNA 183. J Biol Chem 285: 5461-5471.
15. Iliopoulos D, Lindahl-Allen M, Polytarchou C, Hirsch HA, Tsichlis PN, et al. (2010) Loss of miR-200 inhibition of Suz12 leads to polycomb-mediated repression required for the formation and maintenance of cancer stem cells. Mol Cell 39: 761-772.
16. Du C, Liu C, Kang J, Zhao G, Ye Z, et al. (2009) MicroRNA miR-326 regulates TH-17 differentiation and is associated with the pathogenesis of multiple sclerosis. Nat Immunol 10: 1252-1259.
17. Chang KW, Liu CJ, Chu TH, Cheng HW, Hung PS, et al. (2008) Association between high miR-211 microRNA expression and the poor prognosis of oral carcinoma. J Dent Res 87: 1063-1068.
18. Levy C, Khaled M, Iliopoulos D, Janas MM, Schubert S, et al. (2010) Intronic miR-211 assumes the tumor suppressive function of its host gene in melanoma. Mol Cell 40: 841-849.
19. Galardi S, Mercatelli N, Giorda E, Massalini S, Frajese GV, et al. (2007) miR-221 and miR-222 expression affects the proliferation potential of human prostate carcinoma cell lines by targeting p27Kip1. J Biol Chem 282: 23716-23724.
20. Gillies JK, Lorimer IA (2007) Regulation of p27Kip1 by miRNA 221/222 in glioblastoma. Cell Cycle 6: 2005-2009.
21. Hassan MQ, Gordon JA, Beloti MM, Croce CM, van Wijnen AJ, et al. (2010) A network connecting Runx2, SATB2, and the miR-23a~27a~24-2 cluster regulates the osteoblast differentiation program. Proc Natl Acad Sci U S A 107: 19879-19884.
22. Tsuchiya S, Oku M, Imanaka Y, Kunimoto R, Okuno Y, et al. (2009) MicroRNA-338-3p and microRNA-451 contribute to the formation of basolateral polarity in epithelial cells. Nucleic Acids Res 37: 3821-3827.
23. Cervigne NK, Reis PP, Machado J, Sadikovic B, Bradley G, et al. (2009) Identification of a microRNA signature associated with progression of leukoplakia to oral carcinoma. Hum Mol Genet 18: 4818-4829.
24. Huang Y, Chuang A, Hao H, Talbot C, Sen T, et al. (2011) Phospho-DeltaNp63alpha is a key regulator of the cisplatin-induced microRNAome in cancer cells. Cell Death Differ.
25. Shin KH, Bae SD, Hong HS, Kim RH, Kang MK, et al. (2011) miR-181a shows tumor suppressive effect against oral squamous cell carcinoma cells by downregulating K-ras. Biochem Biophys Res Commun 404: 896-902.
26. Li QJ, Chau J, Ebert PJ, Sylvester G, Min H, et al. (2007) miR-181a is an intrinsic modulator of T cell sensitivity and selection. Cell 129: 147-161.
27. Morton SU, Scherz PJ, Cordes KR, Ivey KN, Stainier DY, et al. (2008) microRNA-138 modulates cardiac patterning during embryonic development. Proc Natl Acad Sci U S A 105: 17830-17835.
28. Xue Q, Guo ZY, Li W, Wen WH, Meng YL, et al. (2011) Human activated CD4(+) T lymphocytes increase IL-2 expression by downregulating microRNA-181c. Mol Immunol 48: 592-599.
29. Luedde T (2010) MicroRNA-151 and its hosting gene FAK (focal adhesion kinase) regulate tumor cell migration and spreading of hepatocellular carcinoma. Hepatology 52: 1164-1166.
30. Chen J, Feilotter HE, Pare GC, Zhang X, Pemberton JG, et al. (2010) MicroRNA-193b represses cell proliferation and regulates cyclin D1 in melanoma. Am J Pathol 176: 2520-2529.
31. Nishino J, Kim I, Chada K, Morrison SJ (2008) Hmga2 promotes neural stem cell self-renewal in young but not old mice by reducing p16Ink4a and p19Arf Expression. Cell 135: 227-239.
32. Roush S, Slack FJ (2008) The let-7 family of microRNAs. Trends Cell Biol 18: 505-516.
33. Xu N, Papagiannakopoulos T, Pan G, Thomson JA, Kosik KS (2009) MicroRNA-145 regulates OCT4, SOX2, and KLF4 and represses pluripotency in human embryonic stem cells. Cell 137: 647-658.
34. Cho WC, Chow AS, Au JS (2011) MiR-145 inhibits cell proliferation of human lung adenocarcinoma by targeting EGFR and NUDT1. RNA Biol 8.
35. Elia L, Quintavalle M, Zhang J, Contu R, Cossu L, et al. (2009) The knockout of miR-143 and -145 alters smooth muscle cell maintenance and vascular homeostasis in mice: correlates with human disease. Cell Death Differ 16: 1590-1598.
36. Kent OA, Chivukula RR, Mullendore M, Wentzel EA, Feldmann G, et al. (2010) Repression of the miR-143/145 cluster by oncogenic Ras initiates a tumor-promoting feed-forward pathway. Genes Dev 24: 2754-2759.
